# Twisting Ovaries: Three Cases of Ovarian Torsion

**DOI:** 10.7759/cureus.30496

**Published:** 2022-10-19

**Authors:** Yuchen Duan, Bryce Hoer, Andrew Little

**Affiliations:** 1 Emergency Medicine, AdventHealth East Orlando, Orlando, USA

**Keywords:** ob-gyn, ovarian tumor, emergency medicine training, uterus and ovary, adnexal torsion

## Abstract

Ovarian torsion is a medical emergency that should be considered for every female presenting with abdominal pain. This series consists of three cases of females of varying ages presenting with abdominal pain who were all ultimately diagnosed with ovarian torsion. It highlights the variability in presentation, physical examination, and other factors related to the diagnosis.

## Introduction

Ovarian torsion represents roughly 3% of gynecologic emergencies, with 80% of torsion cases presenting concurrently with some form of ovarian mass ≥ 5 cm [1-4]. Although it can present in females of all ages, it is most common in females of reproductive age. The pathogenesis of torsion involves a physical twisting of the ovary on an attachment point that causes lymphatic, venous, and arterial obstruction progressing to ovarian thrombosis, edema, necrosis, and infarction [4,5]. Although classic textbook presentations of ovarian torsion often paint a clinical picture of “acute-onset pelvic pain in a childbearing age female,” real-world cases can often present with much more mixed and subtle symptomatology that can represent a significant identification challenge. Here, we present three cases of ovarian torsion with non-classical features that represent the broad spectrum of clinical presentations that torsion can manifest.

## Case presentation

Case 1

A 32-year-old female presented to the emergency department (ED) with nausea and pelvic pain for one day. The patient noted that she was currently on her menstrual period with mild vaginal bleeding consistent with her usual cycles. She also noted that her pelvic discomfort was consistent with her usual “menstrual cramps.” Despite most of her clinical symptoms being typical of her previous menstrual periods, the patient noted that she usually does not feel nauseous.

Physical examination was only significant for vague right-sided abdominal tenderness but otherwise unremarkable, with no adnexal tenderness noted on pelvic examination. Furthermore, complete blood count, metabolic panel, urinalysis, and vaginal wet prep were within the normal reference range, and the urine pregnancy test was negative.

**Figure 1:**
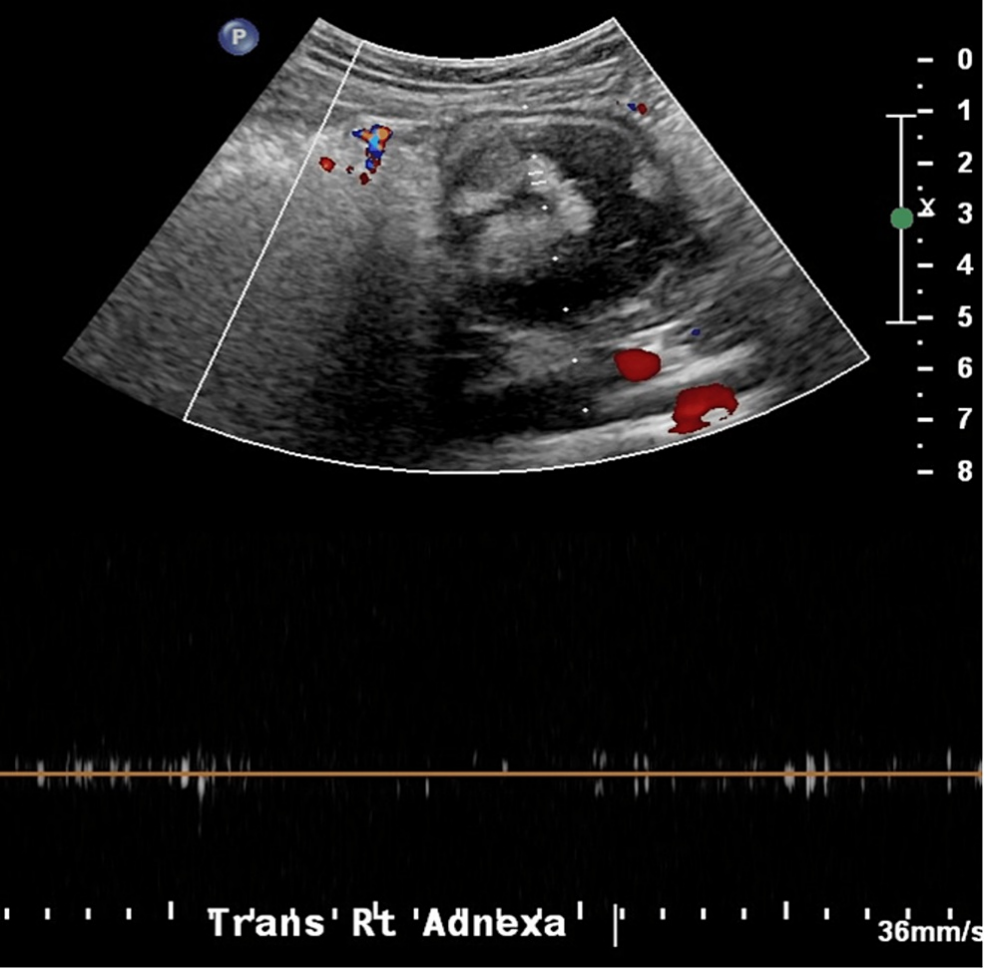
Doppler ultrasound of the right ovary showing a 5-cm teratoma but with normal waveform and color Doppler flow

**Figure 2:**
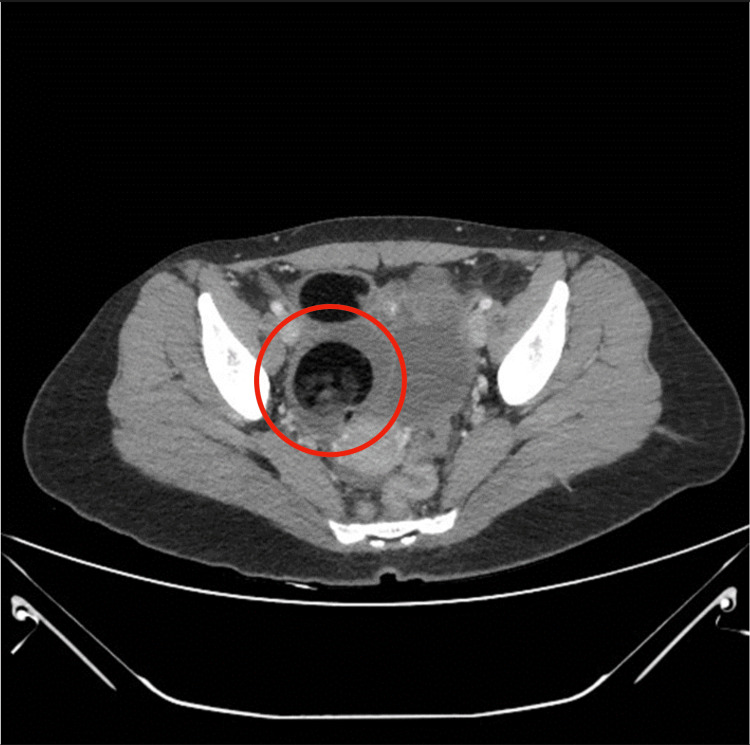
Computed tomography of the abdomen and pelvis showing a 10-cm mass in the right lower quadrant (red circle)

Subsequently, a transvaginal Doppler ultrasound (US) revealed a right adnexal complex echogenic cyst with incomplete visualization favored to represent roughly a 5-cm teratoma but with normal waveform and color Doppler flow noted (Figure 1). Computed tomography (CT) scan of the abdomen and pelvis with contrast was performed to better classify the mass, reporting a 10.2-cm complex solid and cystic tumor projecting in the low central pelvis above the bladder (Figure 2).

During her ED course, the patient had waxing and waning pain with a brief moment when she noted that the pain had entirely resolved. However, as the patient was further observed in the ED, her pain returned suddenly, representing a concern for ovarian torsion secondary to the right 10.2-cm teratoma. The obstetrics and gynecology (OB-GYN) service was consulted, and the patient was immediately transferred for surgical management of the ovarian torsion.

Case 2

An elderly female with a history of hypertension, hyperlipidemia, and renal stones and reported surgical history of “total hysterectomy” presented to the emergency department with a complaint of sudden-onset right lower quadrant pain radiating to the right flank for one day. The patient noted that the location of her pain was reminiscent of her previous renal stone pain, but the character of her pain was different and was “cramping and achy” in character rather than “sharp.”

**Figure 3:**
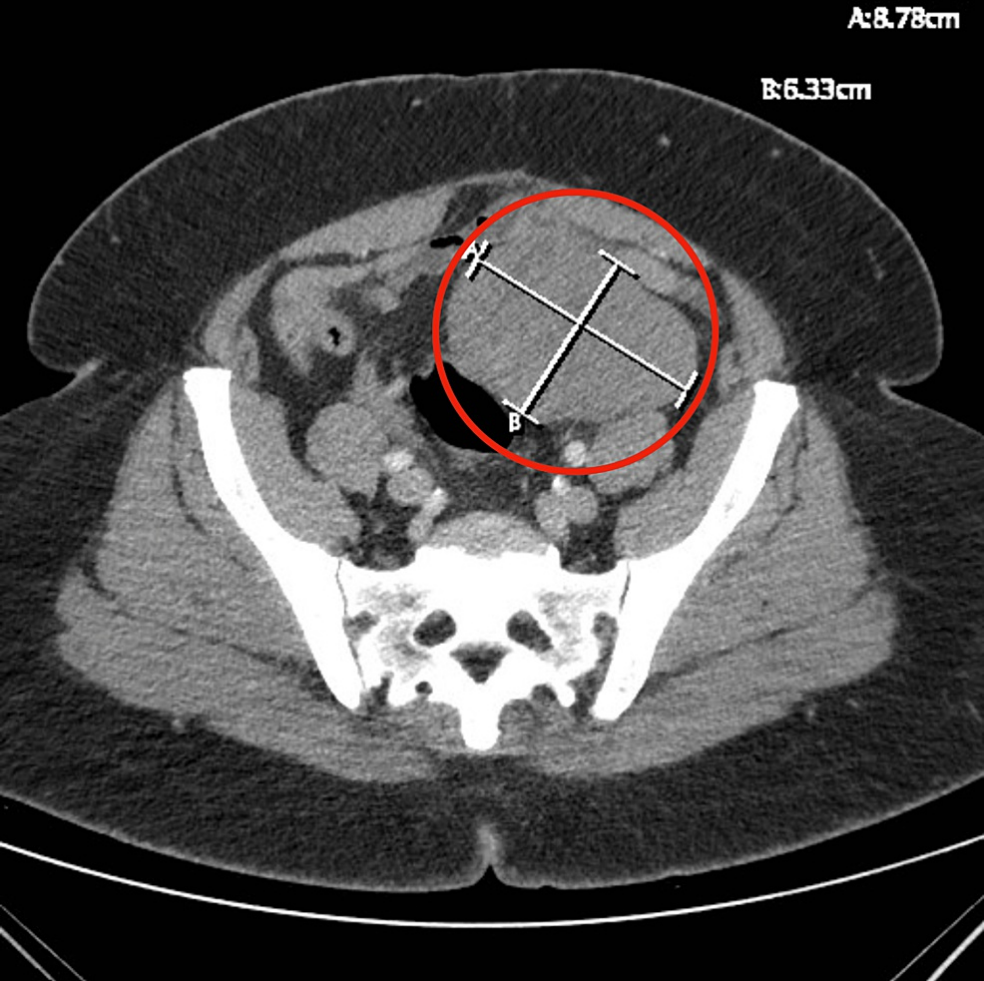
Computed tomography of the abdomen and pelvis showing a large mass in the left lower abdomen concerning for an ovarian mass (red circle)

**Figure 4:**
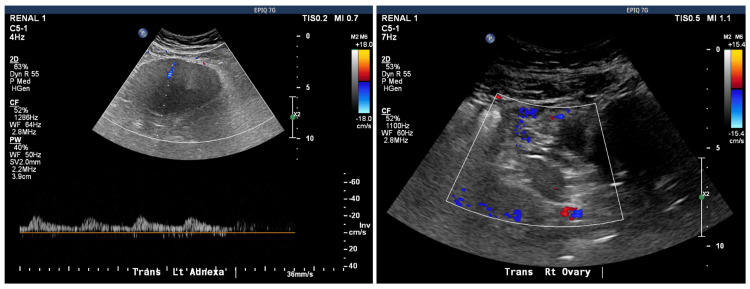
Transabdominal pelvic ultrasound showing no flow to the left adnexal mass versus flow to the right ovary

Physical examination was significant for right lower quadrant tenderness and right flank pain but is otherwise unremarkable. Complete blood count, complete metabolic panel, and urinalysis revealed a mildly elevated white blood count of 11.9, but all were within normal limits. A CT scan of the abdomen and pelvis with contrast showed a soft tissue mass in the left adnexa measuring 8.8 × 6.3 cm but did not show any other intra-abdominal pathology (Figure 3). The patient’s pain continued along the right lower quadrant, and a subsequent transvaginal Doppler ultrasound was performed. The ultrasound did not visualize the uterus or cervix, consistent with the history of hysterectomy, but demonstrated a left adnexal mass with minimal blood flow to the ovary, concerning for an ovarian torsion (Figure 4).

The patient was updated on the findings and noted persistent pain despite IV pain medications. The OB-GYN service was consulted regarding finding a left-sided ovarian tumor with ovarian torsion, and the patient was transferred for operative intervention and further evaluation.

Case 3

Five days post laparoscopic reduction of left ovarian and tubal torsion with left salpingectomy, a 44-year-old female presented to the ED with right-sided abdominal pain for five days. The patient stated that the abdominal pain had persisted since the surgery and was worse on the right side. She denied fever, chills, nausea, vomiting, diarrhea, back pain, vaginal discharge/bleeding, or other symptoms. The patient’s surgical history included a supracervical hysterectomy and right salpingo-oophorectomy 16 years prior. Her physical examination revealed that she had a soft, non-distended abdomen with right-sided tenderness to palpation. The patient had well-healing laparoscopy surgical scars on the right and left abdomen with mild ecchymosis near the surgical incision on the right side of the abdomen.

**Figure 5:**
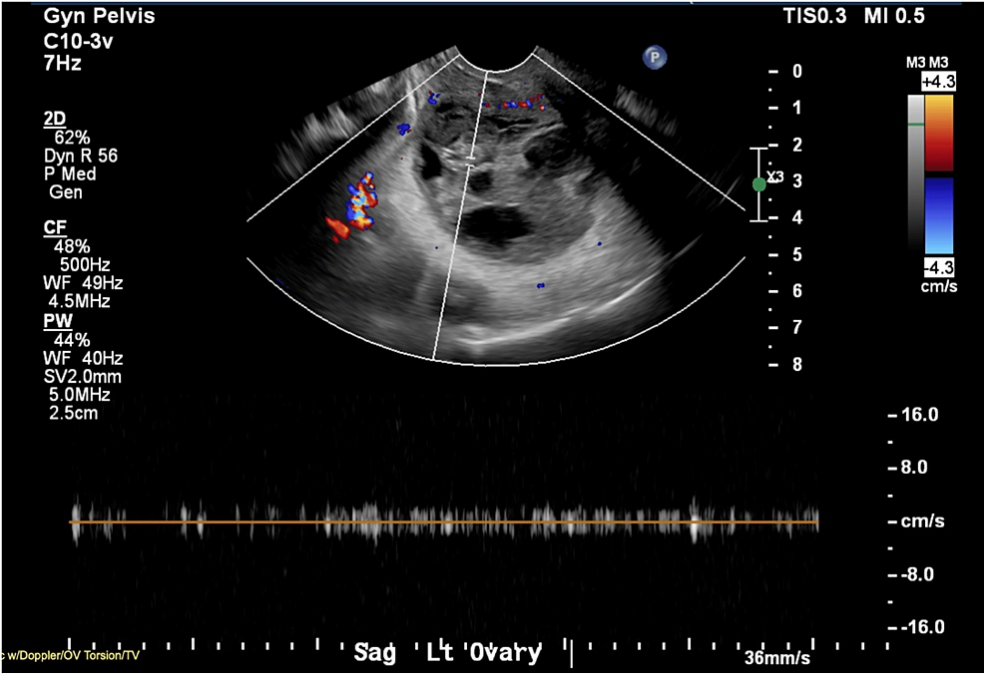
Pelvic ultrasound showing no blood flow to the left ovary

CT of the abdomen and pelvis with contrast showed a size increase of the left ovary, suspicious for recurrent torsion and/or infarction. CT scan prompted pelvic ultrasound with Doppler, which showed a heterogeneous enlarged appearance of the left ovary with no internal flow, consistent with torsion (Figure 5). The patient was taken to surgery emergently with the OB-GYN service for laparoscopic left oophorectomy with reduction and repair of the right quadrant incisional hernia.

## Discussion

The presented cases emphasize the need for emergency medicine and other medical providers to have a high index of suspicion for this ovarian torsion diagnosis in all females with abdominal pain [1]. They also highlight the management principles in ovarian torsions while showcasing that even patients with atypical presentations require workups to find this diagnosis [2]. Although most cases are associated with females of childbearing age, it is integral to remember that by definition, ovarian torsion can present in any female patient with ovaries. It remains a viable differential in evaluating any female with abdominal pain [1].

The presented cases highlight subjects of varying ages and anatomical baselines. In case 1, our patient is a young female with no history of obstetric or gynecologic procedures but with intermittent pain and an enlarged ovary due to a teratoma. Case 2 is an elderly female with an ovarian-sparing hysterectomy. Case 3 is a middle-aged female with a recent reduction of ovarian torsion with salpingectomy. As seen in our second case, we have an elderly female. Although the diagnostic testing of choice for suspected torsion is transvaginal Doppler ultrasound, it is essential to remember that torsion is a diagnosis that can only be confirmed but not ruled out with ultrasound [3]. It has been shown that an average of 40% of torsion cases showed normal Doppler flow on ultrasound. As was the situation in case 1, a combination of a large ovarian mass, continued associated pain, and normal flow on ultrasound was still taken to the operating room for suspected torsion pathology [3].

## Conclusions

It is important to keep a rare diagnosis on any differential because oftentimes, the only thing that is needed to make a rare diagnosis is keeping that possibility in mind. Ovarian torsion is heavily taught as a critical diagnosis in emergency medicine training and is often associated with abdominal pain in young females. However, it is always important to recognize that it can present in any female patient with an existing ovary and, if not recognized in a timely manner, can have devastating consequences on fertility and the patient’s quality of life.
